# Innovative Research for Nutrition- and Climate-Smart Food Systems in Low- and Middle-Income Countries

**DOI:** 10.3390/nu15133020

**Published:** 2023-07-03

**Authors:** Mathilda Freymond, Kesso Gabrielle van Zutphen-Küffer, Klaus Kraemer

**Affiliations:** 1 Sight and Life, P.O. Box 2116, 4002 Basel, Switzerland; 2 Department of Human Nutrition & Health, Wageningen University & Research, 6708 PB Wageningen, The Netherlands; 3 Department of International Health, Johns Hopkins University, Baltimore, MD 2121, USA

The world is off-track to end world hunger, food insecurity, and malnutrition in all its forms by 2030 [1]. In parallel, we are falling behind in international climate targets, and climate change-induced extreme weather events are becoming increasingly prevalent [1,2]. This situation was exacerbated by the global coronavirus disease (COVID-19) pandemic, which further exposed the fragility of our food system by disrupting food production and transportation, leaving even more vulnerable people unable to access safe, nutritious, and affordable foods [3]. As of 2023, 45 million people are on the brink of starvation–enduring famine or famine-like conditions, with women and children bearing the brunt of this crisis [4]. Furthermore, the growing double burden of malnutrition—the coexistence of under- and over-nutrition, is posing a public health burden in many developing regions, largely due to dietary transitions towards more highly processed and less nutrient-dense foods [5,6].

This Special Issue aims to shed light on recent advancements in the fields of nutrition and food systems in low-resource settings. It presents a multi-dimensional perspective, encompassing areas ranging from dietary quality and nutritional assessment to principles for implementing food system innovations and policy. 

Understanding communities’ dietary intake patterns and determining nutritional status are crucial for designing effective food-based approaches to address malnutrition [5]. To enhance the availability and accessibility of diverse, affordable, and nutritious foods, the role of smallholder farmers, women, and small- and medium-sized enterprises (SMEs) should be examined [5,7]. Moreover, the inclusion of nutritious, underutilized crops should be promoted [5]. Schools can also be seen as platforms conducive to the promotion of diverse and nutritious foods, as children and adolescents spend a significant portion of their time in these settings [8]. Schools present an important opportunity to deliver food and nutrition programs, but also health and nutritional education [9]. Furthermore, they can encourage sustainable dietary practices by promoting the consumption of locally grown foods, thereby sustaining local economies and the environment [8,9].

Tackling nutrition-related challenges requires supportive policies, regulations, and implementation frameworks. Comprehensive programs and policies that target the whole supply chain offer a key entry point for nutrition [7]. Repurposing agricultural subsidies, increasing the use of innovations to improve nutrition, and increasing investments in rural infrastructure, communication, and financial support are viable strategies for reshaping policy interventions [7]. 

The successful implementation of local, national, and global food and nutrition policies hinges on understanding the incentives and interests of actors within the food system [7] and requires coordination and collaboration among governments, the private sector, civil societies, NGOs, and other stakeholders [7]. Consumers play a crucial role in driving the transformation of the food system and need to be integrated at every step [10]. By understanding consumer preferences, values, and behaviors, stakeholders can develop sustainable, consumer-centric solutions that meet the evolving needs and preferences of individuals and communities. 

With the escalating double burden of malnutrition, there is an urgent need to generate demand for nutritious diets [11]. However, considering people are not strictly rational decision-makers, interventions should go beyond raising awareness about the benefits of healthy and sustainable eating practices and instead use emotional appeals and social influences [11,12]. Engaging communities as active partners can help in overcoming barriers to change and motivate transformation by communicating and offering benefits that resonate with the target audience [11]. Creating demand for biofortified crops is an example of a food system intervention with the potential to mitigate micronutrient deficiencies [13]. 

Beyond biofortification, other innovations directed towards nutrition and food system transformation have a critical role to play. Emerging food system innovations (FSI) must be developed and implemented holistically to ensure economic, social, and environmental sustainability [14]. Understanding how the different components of the food system interact is indispensable to create synergies toward evidence-based FSI for healthier diets [14]. Recognizing the dynamics within this network requires research across multiple disciplines, such as agriculture, nutrition, economics, environmental science, and policy [14]. This research is necessary to develop evidence-based and sustainable solutions that can address the complex challenges of our food system. Additionally, the continual exchange of information is key to maintaining an environment conducive to active learning and rigorous performance monitoring [15]. Regular sharing, monitoring, and tracking offer food system actors and stakeholders actionable evidence to hold governments, farmers, consumers, and the private sector accountable [15]. This evidence can help to guide and to shape policies and interventions towards informed decisions, ultimately reshaping how food is produced, processed, distributed, and consumed. Such measures can lead us towards not only meeting our global climate commitments, but also meeting food security and nutrition targets. 
